# Marketing and Social Media: A Guide for Libraries, Archives, and Museums

**DOI:** 10.5195/jmla.2021.1315

**Published:** 2021-10-01

**Authors:** Dana Haugh

**Affiliations:** 1 dana.haugh@yale.edu, Harvey Cushing/John Hay Whitney Medical Library, Yale University, New Haven, CT

The second edition of *Marketing and Social Media: A Guide for Libraries, Archives, and Museums* builds on its 2014 publication with updated chapters, new resources, and a heavier focus on social media. Particular attention was paid to the transient nature of social media platforms with the hope that even as they change and evolve, the practices and recommendations will remain useful.

The book begins with a bold declaration: nonprofits have a lot to learn from retailers. At first blush, this is an uncomfortable statement. But the intention is to get the reader thinking about library and museum spaces in terms of the products and services they offer. The authors argue that a “key factor in successful marketing is understanding the needs of customers” and “ultimately [libraries, museums, and archives] desire to maximize customer satisfaction” (p. 1). This, they declare, is why they use the term “customer” throughout the book instead of more common nonprofit terms like “patron,” “user,” or “visitor.” According to Mon and Koontz, those who visit and engage with your institution are not kindly benefactors, but customers whose needs, preferences, and expectations directly influence your organization's success or failure. While not all retail practices are applicable to nonprofits, the authors outline five areas where retail concepts can be applied to the nonprofit sector, such as offering products and services based on demand and providing diverse channels of delivery.

The monograph comprises fifteen chapters, each building on the last to deliver a comprehensive roadmap for navigating nearly every aspect of marketing. The monograph's value is its ability to translate these typically profit-hungry practices into strategies suitable for library and museum spaces. After an overview of core marketing concepts and terms, the second chapter details different types of marketing models and provides recommendations for developing a mission statement. A mission statement that speaks to a library's or museum's purpose is key as it will directly inform an organization's strategic planning and marketing goals. From there, the book introduces foundational marketing practices such as collecting data through environmental scans, conducting a SWOT analysis, and strategies for understanding your stakeholders. In chapters 6 and 7, the authors dive into the four-step marketing model introduced briefly in chapter 1. Chapters 8–14 cover each component of the model in greater detail and include sections on how you can apply that step to social media. For instance, chapter 8 covers characteristics and strategies for market segmentation and then translates these recommendations into actionable ways you can segment audiences on platforms like Twitter and Reddit. The final chapter, “From the Social Media Manager's Perspective,” walks new social media managers through social media benchmarking, creating content and organizational calendars, developing policies and documentation, analyzing and optimizing your social media presence, and assessing the success and impact of your social media presence. An annotated bibliography that highlights resources by topic (marketing blogs, professional websites, etc.) is located at the end of the book.

The discussion questions at the end of each chapter are perhaps the most valuable content in this monograph. These prompts invite the reader to consider the chapter's topic in relation to their own library or museum and respond accordingly. Though helpful to reiterate the main points of each chapter and provide a practical application, the questions alone would be enough to help a library or museum develop comprehensive marketing plans, mission statements, strategic goals, and promotional campaigns.

Overall, this title is extremely well researched, thorough in its coverage, and provides excellent real-life applications for putting theory into practice.

